# Negative autoimmunity in a Spanish pediatric cohort suspected of type 1 diabetes, could it be monogenic diabetes?

**DOI:** 10.1371/journal.pone.0220634

**Published:** 2019-07-31

**Authors:** Inés Urrutia, Rosa Martínez, Itxaso Rica, Idoia Martínez de LaPiscina, Alejandro García-Castaño, Anibal Aguayo, Begoña Calvo, Luis Castaño, on behalf of The Spanish Pediatric Diabetes Collaborative Group

**Affiliations:** 1 Biocruces Bizkaia Health Research Institute, Cruces University Hospital, UPV-EHU, Bizkaia, Spain; 2 CIBERDEM, CIBERER, Instituto de Salud Carlos III, Madrid, Spain; 3 Pediatric Endocrinology Service, Cruces University Hospital, Osakidetza, Bizkaia, Spain; Brown University Warren Alpert Medical School, UNITED STATES

## Abstract

**Objective:**

Monogenic diabetes can be misdiagnosed as type 1 or type 2 diabetes in children. The right diagnosis is crucial for both therapeutic choice and prognosis and influences genetic counseling. The main objective of this study was to search for monogenic diabetes in Spanish pediatric patients suspected of type 1 diabetes with lack of autoimmunity at the onset of the disease. We also evaluated the extra value of ZnT8A in addition to the classical IAA, GADA and IA2A autoantibodies to improve the accuracy of type 1 diabetes diagnosis.

**Methods:**

Four hundred Spanish pediatric patients with recent-onset diabetes (mean age 8.9 ± 3.9 years) were analyzed for IAA, GADA, IA2A and ZnT8A pancreatic-autoantibodies and HLA-DRB1 alleles. Patients without autoimmunity and those with only ZnT8A positive were screened for 12 monogenic diabetes genes by next generation sequencing.

**Results:**

ZnT8A testing increased the number of autoantibody-positive patients from 373 (93.3%) to 377 (94.3%). An isolated positivity for ZnT8A allowed diagnosing autoimmune diabetes in 14.8% (4/27) of pediatric patients negative for the rest of the antibodies tested. At least 2 of the 23 patients with no detectable autoimmunity (8%) carried heterozygous pathogenic variants: one previously reported missense variant in the *INS* gene (p.Gly32Ser) and one novel frameshift variant (p.Val264fs) in the *HNF1A* gene. One variant of uncertain significance was also found. Carriers of pathogenic variants had HLA-DRB1 risk alleles for autoimmune diabetes and clinical characteristics compatible with type 1 diabetes except for the absence of autoimmunity.

**Conclusion:**

ZnT8A determination improves the diagnosis of autoimmune diabetes in pediatrics. At least 8% of pediatric patients suspected of type 1 diabetes and with undetectable autoimmunity have monogenic diabetes and can benefit from the correct diagnosis of the disease by genetic study.

## Introduction

Type 1 diabetes (T1D) is an organ-specific autoimmune disorder caused by the destruction of insulin-producing pancreatic β-cells leading to an absolute insulin deficiency. Although T1D can be diagnosed at any age, it is one of the most common chronic diseases of childhood. Peaks in presentation occur between 5–7 years of age and at or near puberty [1]. The presence of autoantibodies against several pancreatic islet molecules in response to the autoimmune process is, to date, the best predictive and diagnostic marker for T1D [2]. The autoantibodies that have been of most interest from a clinical and research perspective are islet-cell cytoplasmic autoantibodies (ICA), those against the 65kD isoform of glutamic acid decarboxylase (GADA), the pancreatic tyrosine phosphatase-like molecule IA-2 (IA-2A) and insulin autoantibodies (IAA). More recently, Zinc transporter 8 protein islet autoantibodies (ZnT8A) have also been identified [3]. Nevertheless, not all patients with suspected T1D show evidence of autoimmunity on the basis of the above-mentioned markers [4].

The trigger of the autoimmune process associated with T1D is determined by complex interactions between several genetic loci (nearly 40 loci described so far) and environmental factors [1]. Susceptibility to and protection against the development of autoimmune diabetes are mainly associated with the highly polymorphic sequences of the HLA class II genes on chromosome 6p21. In Caucasians, HLA haplotypes DRB1*03:01-DQA1*05:01-DQB1*02:01 and DRB1*04-DQA1*03:01-DQB1*03:02 confer the greatest susceptibility, while the DRB1*15:01-DQA1*01:02-DQB1*06:02 haplotype provides disease protection [5].

Monogenic diabetes (MD) is a clinically and genetically heterogeneous disease that includes maturity onset diabetes of the young (MODY) and infancy-onset and neonatal diabetes mellitus, which are characterized by functional defects of pancreatic β-cells resulting in insulin deficiency and moderate to severe hyperglycemia in early life [6]. It accounts for at least 1–2% of all cases of diabetes. MODY, the most common type of monogenic diabetes, is an autosomal-dominant form of non-autoimmune diabetes, typically diagnosed before 25 years of age. More than 12 different genes have been associated with MODY. Pathogenic variants in *GCK* and *HNF1A* genes account for approximately 80% of all MODY cases followed by *HNF4A* and *HNF1B* genes representing about 10% and 6% respectively, although percentages can differ dramatically country-to-country due to different recruitment biases. Pathogenic variants in the remainder of the genes are rare forms of MODY [7].

Given that the clinical features of MD are often non-specific, it is estimated that around 80% of MD cases remain undiagnosed or are misdiagnosed as type 1 or type 2 diabetes [7]. Identification of the correct etiology of diabetes is crucial for clinical management, therapeutic choice and prognosis, as well as for genetic counseling, when applicable [8].

The main objective of this study was to search, in a cohort of Spanish pediatric patients suspected of T1D, whether a monogenic form of diabetes could be identified in cases with absence of immunological markers at the onset of the disease. We also aimed to assess the further value of ZnT8A screening in addition to the classical IAA, GADA and IA2A autoantibodies for the diagnosis of autoimmune diabetes.

## Methods

### Patients

We recruited 400 unrelated pediatric patients with recent-onset type 1 diabetes diagnosed according to the International Society for Pediatric and Adolescent Diabetes (ISPAD) criteria [9] who were less than a week on insulin replacement therapy. Participants were recruited from seven referral hospitals in Spain between 2012 and 2017 (mean onset age 8.9 ± 3.9 years, 47.3% female). In all cases HLA-DRB1 alleles and IAA, GADA, IA2A and ZnT8A at diagnosis were analyzed. It was considered positive autoimmunity to have at least one positive antibody. Clinical data collected from all patients at diabetes onset included: age, presence of diabetic ketoacidosis (DKA) according to the ISPAD criteria [10] and family history of diabetes (Table 1 and S1 Table).

**Table 1:** Characteristics of the population included in the study according to antibody status.

	Antibody-positive (n = 377)	Antibody-negative (n = 23)	*p*-value
Gender (% female)	48.0	34.8	n.s.^a^
Age of onset (years)	8.9 ± 3.9	9.6 ± 4.2	n.s.^c^
1^st^ degree relatives with any DM	17.2	13.0	n.s.^b^
Presence of DKA	40.8	18.2	0.035^a^
HLA-DRB1* 0 risk alleles	8.0	8.7	n.s.^b^
HLA-DRB1* 1 risk allele	42.4	43.5	n.s.^a^
HLA-DRB1* 2 risk alleles	49.6	47.8	n.s.^a^

Age is shown as mean ± SD. Rest of data is shown as %. 1^st^ degree relatives: parents and/or siblings (Ab+ n = 372; Ab- n = 23). DKA: Diabetic Ketoacidosis (Ab+ n = 377; Ab- n = 22). HLA-DRB1 risk alleles are defined based on the report [11]: 0 risk alleles (no DR3 no DR4); 1 risk allele (DR3 or DR4); 2 risk alleles (DR3/3, DR4/4 or DR3/4).

^a^ Pearson´s chi-square test

^b^ Fisher`s exact test

^c^ Mann-Whitney U-test

n.s.: no significant differences (p>0.05).

In cases with absence of autoimmunity, more detailed clinical data recorded at diagnosis and at the last check-up was added. These included: body mass index (BMI) expressed as z-score for children according to the 2010 Spanish growth charts [12], duration of diabetes symptoms (polydipsia, polyphagia, polyuria, weight loss and/or blurred vision), analytical data and insulin dose (Table 2). The study was approved by the corresponding Clinical Research Ethics Committee and written informed consent was obtained from all subjects and/or their parents.

**Table 2:** Clinical characteristics and HLA-DRB1 typing of the 23 patients with suspected T1D and negative autoinmmunity.

Patient ID	Background		Clinical features at diagnosis								Clinical features at last check-up					HLA-DRB1
	Gender	1^st^ degree relatives with DM	Age at onset (years)	BMI z-score	DM Symptoms duration (days)	DKA	Glucose (mmol/L)	HbA1C (%)	C-Peptide (nmol/L)	Insulin dose (U/Kg/day)	Time of evolution (years)	BMI z-score	HbA1C (%)	Insulin dose (U/Kg/day)	Other autoinmune disease	Allele1/Allele 2
1	F	0	9.3	-1.29	30	No	37.91	>15.0	< 0.02	0.85	5.6	0.14	7.7	0.53	No	0301 / 0301
2	M	0	14.7	2.72	20	No	13.82	13.0	0.57	0.75	4.2	2.29	6.5	0.32	Thyroiditis	0102 / 1101
3	F	Mother	14.3	0.41	90	No	15.54	12.5	0.71	0.80	1.4	1.69	9.0	1.70	No	0404 / 0802
4	M	0	7.0	-0.74	14	Yes	23.42	11.7	0.10	0.90	5.0	-0.44	8.7	0.80	No	0403 / 1102
5	M	0	13.3	1.59	15	No	19.81	11.1	0.16	0.85	2.1	0.63	6.4	0.80	No	0301 / 1601
6	M	0	16.0	-0.12	30	No	28.80	11.0	0.10	0.65	2.7	-0.18	6.5	0.17	No	0301 / 0301
7	M	0	8.1	-2.02	21	Yes	41.96	13.1	N/D	1.00	2.2	-1.29	6.0	0.46	No	0301 / 1601
8	M	0	15.9	3.18	60	No	16.65	12.7	0.34	0.90	4.8	N/D	7.5	N/D	N/D	0404 / 0901
9	F	0	9.3	0.64	15	No	21.92	11.5	0.41	0.81	4.7	0.35	7.5	0.82	No	0301 / 0405
10	F	0	10.8	0.37	6	No	11.99	13.0	0.33	0.68	2.9	0.51	6.7	0.50	No	0301 / 0403
11	M	N/D	5.7	-0.41	5	No	8.82	8.7	0.38	0.33	2.9	-0.63	8.1	0.41	No	0405 / 1401
12	M	0	7.3	-0.39	3	No	12.88	5.9	0.61	0.40	2.9	-0.05	7.6	1.00	No	0301 / 0701
13	M	0	8.0	-1.25	7	No	25.20	13.0	0.10	1.02	2.7	-0.63	6.9	0.77	No	0301 / 0801
14	F	0	13.8	-0.41	180	Yes	>27.75	17.5	N/D	1.27	2.3	-0.42	11	0.49	No	0101 / 0401
15	M	0	7.4	-0.37	7	No	18.04	10.8	0.29	0.97	2.3	-0.20	8.2	0.84	No	0401 / 0701
16	M	0	9.9	-0.46	60	No	13.93	11.0	0.13	0.90	2.8	-0.63	6.7	0.79	Thyroiditis	03 / 04
17	F	0	8.5	-0.70	30	No	51.84	17.2	N/D	1.23	1.7	0.17	7.6	0.87	No	0301 / 0405
18	M	Mother	12.0	0.35	7	No	11.27	8.9	0.51	0.62	0.8	0.02	6.4	0.00	No	0301 / 0402
19	M	0	14.4	0.51	30	No	18.54	10.5	0.57	0.69	0.4	0.46	5.8	0.17	No	0301 / 0301
20	M	Mother	0.8	-1.16	7	Yes	18.34	8.8	0.04	0.76	0.7	-0.10	8.1	0.61	Coeliac disease	0301 / 0404
21	F	0	2.2	-0.47	21	N/D	24.70	11.8	0.05	1.20	0.3	2.74	7.1	0.74	No	0301 / 0401
22	M	0	5.6	1.14	7	No	20.59	11.0	0.08	0.72	0.8	0.14	7.1	0.60	No	0301 / 0401
23	F	0	6.3	-0.68	60	No	40.13	13.6	0.15	1.20	0.9	0.23	8.4	0.56	No	0301 / 0404

F: Female. M: Male. 1^st^ degree relatives: parents and/or siblings. BMI z-score: Body mass index expressed as z-score for children. Symptoms of DM: At least 2 of the classic symptoms associated with disease onset, that is, polydipsia, polyphagia, polyuria, weight loss and/or blurred vision. DKA: Diabetic Ketoacidosis. Normal range for: Glucose 3.72–6.1 mmol/L; Fasting Serum C-Peptide 0.26–1.39 nmol/L; HbA1C 4.8–6.2%. N/D: No data.

### Antibody analyses

Pancreatic autoantibodies (IAA, GADA, IA2A and ZnT8A) were determined in serum at diagnosis, using previously described standardized radio-assays [13]. IAA were determined using a competitive fluid-phase radio-assay which uses [^125^I]-labelled, recombinant human insulin (PerkinElmer Inc., Waltham, MA, USA) as antigen. GADA, IA2A and ZnT8A were determined by means of standardized radio-binding assays using *in vitro* transcribed and translated [^35^S]-labelled recombinant human full-length GAD65, IA2ic (amino acids 605–979) and ZnT8 antigen. The ZnT8A assay simultaneously measures autoantibodies against both variants of the COOH-terminal domain (Arg325/Trp325). Antibody results for GADA, IA2A and ZnT8A are expressed as a semi-quantitative index calculated using a dilution curve of a positive sample. All cut-off values were set at the 99th percentile of the control population. Our laboratory has participated in different islet autoantibody standardization program (IASP) workshops, the last one in 2018. Specificity was 100% for all four antibody assays and sensitivity was 65% for IAA, 68% for GADA and 62% for IA2A and ZnT8A.

### Genetic analyses

DNA extraction was performed using the QiAamp DNA blood kit (Qiagen, Hilden, NRW, Germany). DNA quality and quantification was assessed using both NanoDrop ND-1000 (Thermo Fisher Scientific, Waltham, MA, USA) and Qubit 2.0 Fluorometer (Thermo Fisher Scientific) consistent with the manufacturers' instructions.

#### HLA-DRB1 typing

Polymerase chain reaction sequence-specific oligonucleotide method (PCR-SSO) combined with Luminex technology was carried out using the LABType RSSOH2B1 (HLA-DRB1-HD) commercial kit (One Lambda, Inc., Canoga Park, CA, USA). All procedures were performed according to the manufacturers' instructions. HLA-DRB1 risk alleles for T1D were defined based on the previous report published by our group [11].

#### Genetic screening

Genetic testing was performed by next generation sequencing (NGS). A customized gene panel was designed with the Ion AmpliSeq Designer tool v.4.4.8 (www.ampliseq.com). The gene panel comprised the 3' and 5' UTR regions, promoters, the entire coding region and exon-intron boundaries (± 50 bp) of 12 known genes related to monogenic diabetes, including the most frequent genes, such as *GCK*, *HNF1A*, *HNF4A*, *HNF1B*, *INS*, *ABCC8*, *KCNJ11* and the infrequent ones, *PDX1*, *NEUROD1*, *KLF11*, *PAX4* and *BLK*. The target size was 69,220 bp (272 amplicons from 125 to 375 bp in size) with a theoretical coverage of 99.13% for the targeted regions.

Libraries were prepared in three pools per patient with the Ion Ampliseq Library kit 2.0 (Thermo Fisher Scientific) according to the manufacturer´s protocol. Sequencing was performed on a PGM Ion Torrent NGS sequencer using the Ion PGM Hi-Q View Sequencing kit (Thermo Fisher Scientific) and an Ion 316 chip to obtain an average coverage depth of 100 reads per base. Sequence alignment and variant calling were performed using Torrent Suite Software v.5.0.4. Resulting aligned reads (BAM files) and variant calling files (VCF files) were then transferred to Ion Reporter Software v.5.10.0 for variant annotation. Variants with low quality (Phred-like score ≤ 30 associated with a *p*-value > 0.001) and Minor Allele Frequency (MAF) > 0.01 in population databases were excluded. The minimal depth per base established to validate the sequence was 20 reads and any area of interest that did not reach at least 20 reads was sequenced by Sanger (ABI 3130xl Genetic Analyzer, Thermo Fisher Scientific). The average coverage depth achieved per base and per patient was 508 reads (from 885 to 218 with a base coverage uniformity of 92%) and 98% of targeted bases were covered by more than 20 reads.

For the validation of this panel we included independent DNA samples from 29 patients with 64 previously sequenced variants. The analysis showed a sensitivity of 98.5% and a specificity of 96.5% for point variations and small indels (from 1 to 15 bases).

All variants of interest were confirmed by Sanger sequencing using the Big Dye Terminator v.3.1 Cycle Sequencing Kit (Thermo Fisher Scientific) and the ABI 3130xl DNA sequencing system (Thermo Fisher Scientific). When possible, parents and family members of positive patients were analyzed.

#### Variant classification

The pathogenicity of rare variants detected (MAF ≤ 0.01) was determined according to the recommendations of the American College of Medical Genetics (ACMG) for variant classification and reporting [14]. These guidelines classify variants into five categories: pathogenic, likely pathogenic, variant of unknown significance (VUS), likely benign and benign. The ACMG criteria for variant classification are based on a set of different evaluation fields. Population data was determined from public genomic databases (1000 Genomes Project, GnomAD and dbSNP). Other criteria to consider were based on the type of variant (eg. frameshift, nonsense and essential splice variants) and on clinical, functional and genotype-phenotype data from the literature and disease databases (Human Gene Mutation Database Professional, PubMed). If such variants had not been reported previously, they were evaluated to predict their possible functional significance using *in silico* prediction tools such as SIFT (http://sift.jcvi.org/), PolyPhen2 (http://genetics.bwh.harvard.edu/pph2/), PROVEAN (http://provean.jcvi.org/index.php), Mutation Taster (http://www.mutationtaster.org/), Panther (http://pantherdb.org/tools/csnpScoreForm.jsp), MutPred2 (http://mutpred.mutdb.org/) and SNPs&GO (https://snps-and-go.biocomp.unibo.it/snps-and-go/). Rare variants were considered to be a VUS if the available information had limited or contradictory evidence for pathogenicity.

#### MLPA analysis

Patients without pathogenic variants detected by NGS were analyzed by Multiplex Ligation-dependent Probe Amplification (MLPA) to identify partial and whole gene deletions or duplications of *GCK*, *HNF1A*, *HNF4A* and *HNF1B* genes, using the SALSA MLPA MODY P241 Kit (MRC-Holland, Amsterdam, The Netherlands) according to the manufacturer’s instructions. Fragments were separated by capillary electrophoresis (ABI 3130xl Genetic Analyzer) and analyzed using Gene-Mapper, v.4.0 software (Thermo Fisher Scientific).

### Statistical analysis

Statistical analysis was carried out using SPSS software (v.21; SPSS Inc., Chicago, IL). Quantitative variables were expressed as means and standard deviations and qualitative variables as frequencies and percentages. The Mann-Whitney U test was used to compare quantitative variables. Frequencies were compared using Pearson´s chi-square analysis and Fisher’s exact test when necessary. The significance level was defined as *p* < 0.05.

## Results

### Immunological data

As shown in Table 3, prevalence of GADA, IA2A, IAA and ZnT8A in the 400 new onset pediatric T1D patients was 73.3, 67.3, 64.8 and 57.3%, respectively. In 94.3% of the patients an autoimmune response against the pancreatic β-cells was detected and 82.8% of them had two or more positive antibodies. ZnT8A testing increased the number of autoantibody-positive patients from 373 (93.3%) to 377 (94.3%) and allowed us to diagnose T1D in 14.8% (4/27) of pediatric patients who were negative for the rest of the antibodies tested. Finally, 23 pediatric patients (23/400, 5.7%) with clinical T1D diagnosis had negative autoimmunity.

**Table 3:** Patients with positive autoimmunity according to the number of antibodies evaluated.

1 antibody	Patients with positive autoinmmunity	%
ZnT8A	229	57.3
IAA	259	64.8
IA2A	269	67.3
GADA	293	73.3
2 antibodies		
IA2A, ZnT8A	316	79.0
IAA, IA2A	333	83.3
IAA, ZnT8A	337	84.3
GADA, ZnT8A	338	84.5
GADA, IAA	351	87.8
GADA, IA2A	361	90.3
3 antibodies		
IAA, IA2A, ZnT8A	352	88.0
GADA, IA2A, ZnT8A	367	91.8
GADA, IAA, ZnT8A	370	92.5
GADA, IAA, IA2A	373	93.3
4 antibodies		
GADA, IA2A, IAA, ZnT8A	377	94.3

In all patients (n = 400) IAA, GADA, IA2A and ZnT8A were analyzed at diagnosis. Data shows the results of the different combinations of one or more antibody detection assays. It is considered positive autoimmunity to have at least one antibody-positive.

### Clinical data and HLA-DRB1 typing

The frequency of ketoacidosis at diagnosis was higher in patients with positive autoimmunity compared with patients who did not show autoimmunity (40.6% *vs*. 18.2%, *p* = 0.036). There were no significant differences in the age at diagnosis, the family history of diabetes and the presence of HLA-DRB1 risk alleles for T1D between these two groups of patients (Table 1).

The clinical characteristics and HLA-DRB1 typing of the 23 autoimmune negative patients are detailed in Table 2. In these 23 patients the mean onset age was 9.6 ± 4.2 years and 34.8% were female. At the time of diagnosis, the mean BMI z-score was 0.02 ± 1.24, only two patients had obesity. All patients had hyperglycemia with a low mean C-peptide value of 0.28 ± 0.22 nmol/l and symptoms of diabetes at disease onset lasting from 3 days to 6 months. However, ketoacidosis was identified at clinical presentation in only 18.2% (4/22) of them. HLA-DRB1 risk alleles for T1D were absent in two patients and the rest had 1 or 2 risk alleles.

### Genetic data

A customized panel of 12 MODY genes was tested in 23 patients with negative autoimmunity. The genetic screening revealed a total of five different rare variants (MAF < 0.01) in 6 patients; all variants were present in a heterozygous state (Table 4). Two of them were classified as pathogenic, representing 8% (2/23) of pediatric patients with suspected T1D and negative autoimmunity. No partial or whole gene deletions or duplications were detected.

**Table 4:** Rare genetic variants in MD genes identified in patients with suspected T1D and negative autoimmunity.

Patient ID	Gene	Exon	Nucleotide change	AA change	Type	dbSNP	MAF_ALL GnomADe	Variant effect	Ref.
11	INS	2	c.94G>A	p.Gly32Ser	Missense	rs80356664	0	Pathogenic	[15–19]
18	HNF1A	4	c.789dupT	p.Val264fs	Frameshift	None	0	Pathogenic	
14	HNF1B	4	c.1006C>G	p.His336Asp	Missense	rs138986885	0.0002	VUS	[20–22]
17	HNF1A	1	c.92G>A	p.Gly31Asp	Missense	rs137853247	0.001	Likely benign	[23–27]
9 & 22	HNF1B	1	c.226G>T	p.Gly76Cys	Missense	rs144425830	0.001	Likely benign	[27–31]

Reference sequences: *INS*, NM_000207.2; *HNF1A*, NM_000545.5; *HNF1B*, NM_000458.2. dbSNP: Single Nucleotide Polymorphism Database. MAF_ALL in GnomADe: Maximum Allele Frequency of the variant in the Exome Genome Aggregation Database. VUS: Variant of Uncertain Significance

We also performed the genetic study in the 4 patients with only ZnT8A positive autoimmunity. None of them were found to have any variant suspected of monogenic diabetes.

The p.Gly32Ser pathogenic variant in the *INS* gene (patient 11) is known to cause proinsulin misfolding [15] and has been previously reported in patients with permanent neonatal diabetes [16] and also in patients with diabetes onset during infancy, childhood or adulthood [17–19]. Parental samples were unavailable for genetic analysis. We also identified a novel pathogenic variant (p.Val264fs) in the *HNF1A* gene (patient 18) that, as far as we know, has not been published. This variant involves the thymine duplication at nucleotide 789, resulting in a frameshift that generates a premature stop codon at position 53 of the new reading frame. Thus, it is expected to result in a truncated protein with loss of normal function. The variant co-segregates with early-onset diabetes in the relatives of this family. It was identified in the younger brother of the proband who had been diagnosed later with diabetes at the age of 10 years. His mother, who was diagnosed primarily with gestational diabetes and subsequently with diabetes, also carries the same variant.

In our cohort, three other rare variants were found. One VUS variant in the *HNF1B* gene (p.His336Asp) was found in patient 14. Parental samples were unavailable for genetic analysis and there is no information about extra-pancreatic complications. Additionally, *in silico* analyses were inconsistent. Two other rare variants have recently been re-classified as likely benign variants. The p.Gly31Asp variant in the *HNF1A* gene was found in patient 17. Parental analysis revealed that the variant is carried by her asymptomatic mother and *in silico* analysis predicted contradictory results. Finally, the p.Gly76Cys variant in the *HNF1B* gene is carried by patients 9 and 22. In both cases the variant was inherited from asymptomatic parents who had no apparent extra-pancreatic complications associated with *HNF1B*. All the *in silico* algorithms predicted a deleterious impact.

## Discussion

Different studies of newly diagnosed T1D patients indicate that 6–18% of children and adolescents with clinical T1D do not show evidence of humoral islet autoimmunity at disease onset [4,32,33]. Our study, with 6% (23/400) of pediatric patients with clinical diagnosis of T1D and undetectable autoimmunity, corroborates these results.

The molecular genetic screening performed in these autoimmune negative patients showed five different rare variants. At least two of them are clearly pathogenic alterations responsible for monogenic diabetes: p.Gly32Ser in the *INS* gene and p.Val264fs in the *HNF1A* gene. Consequently, monogenic diabetes was identified in at least 8% (2/23) of pediatric patients with suspected T1D and negative autoimmunity. Other studies with different approaches, have estimated a prevalence comparable to that of our cohort [34–37]. Furthermore, our study provides additional evidence that pathogenic variants in *INS* and *HNF* genes play critical roles in childhood-onset patients with antibody-negative but insulin-requiring diabetes. We did not find any pathogenic variant in the *GCK* gene despite its major role in MODY. It is not a surprising result because heterozygous *GCK* pathogenic variants result in moderate diabetes that does not match the typical clinical features of T1D onset on which our study is focused.

In the *HNF1A*-MODY case (p.Val264fs), three family members carried the same pathogenic variant, the proband, his mother and the younger brother. The genetic finding allowed us to adjust the treatment of the mother with oral anti-diabetic agents and to prescribe sulfonylureas for the younger brother from the onset of the disease. We simultaneously changed the treatment of the proband from insulin to sulfonylureas. Mother and younger brother demonstrated a successful response to sulfonylureas. Due to the non-compliance with therapy, the proband exhibited unstable diabetes control.

Regarding the p.His336Asp variant in the *HNF1B* gene, it is unclear if it is pathogenic. Published data do not show clear evidence of pathogenicity [20,21] and it has been classified as a variant of uncertain significance in a recent report [22]. The other two rare variants, p.Gly31Asp in the *HNF1A* gene and p.Gly76Cys in the *HNF1B* gene, have been recently re-classified as likely benign polymorphisms [27]. Although the p.Gly31Asp variant has been previously described in the literature associated with monogenic diabetes [23], the pathogenicity of this variant is currently questioned based on allele frequency data in the general European population [24,25] and on functional studies that do not demonstrate a clear impairment of protein functionality [26]. In addition, the family study shows the variant is inherited from an unaffected mother. Therefore, based on this information, we interpret p.Gly31Asp as a likely benign variant. The situation is similar concerning the p.Gly76Cys variant in the *HNF1B* gene which, although previously described in the literature associated with monogenic diabetes [28], has been recently found at a frequency of 0.5% in a healthy Spanish population [29] and greater than 8% in the North African population [30,31]. This allele frequency is higher than that expected based on the estimated prevalence of monogenic diabetes in the population. Moreover, the family study shows that unaffected relatives carry the variant, so based on this information we interpret p.Gly76Cys as a likely benign variant.

The clinical and analytical data of the patients with non-autoimmune diabetes in our cohort support the diagnosis of T1D and, except for the absence of autoantibodies, do not specifically suggest monogenic diabetes. Low fasting serum C-peptide values at the onset of the disease (less than 0.2 nmol/l) denote low endogenous insulin production and correlate with T1D [38]. In our cohort, 55% (11/20) of the children with negative autoimmunity had low C-peptide levels in conjunction with hyperglycemia at diagnosis. The presence of slightly higher levels in the rest of the patients might be related to a greater pancreatic reserve at the onset of the disease. On the other hand, there are only two patients with obesity (patient 2 and 8) who might be suspected of having T2D. However the likelihood of T2D has been discarded as it is a rare disease in pediatrics in Europe [39,40]. In addition, both cases are Caucasian, with healthy parents, clear symptoms of diabetes at diagnosis, not too high C-peptide levels and no clinical or analytical evidence of insulin resistance. Furthermore, one of them has autoimmune thyroiditis. Therefore, although the absence of positive pancreatic-autoantibodies suggests monogenic diabetes, a diagnosis of T1D in our cohort should not be definitively ruled out. There could be other autoantibodies, still not identified, that could have contributed to the development of the autoimmune process in these patients and that could be signaled by positivity to islet cell antibodies (ICA) [32,41]. Additionally, some mildly positive cases could not be identified by the assays. This error is minimized in our study by the combined measurement of four pancreatic-antibodies for the diagnosis, achieving a 94.3% autoimmunity detection rate at the onset of the disease.

In our cohort, the addition of the ZnT8A test to the traditional set of diabetes associated autoantibodies has made it possible to identify autoimmunity in four patients who were ZnT8A positive and otherwise antibody-negative. This represents a non-negligible percentage, close to 15% of patients (4/27), who were diagnosed properly as T1D. The absence of pathogenic variants in the four patients with only ZnT8A positive, supports recently published data confirming ZnT8A as a marker for excluding patients from genetic testing for monogenic diabetes [42,43].

Although autoantibodies are so far considered as the best biomarker to discriminate MODY from T1D, we cannot ensure that there is no monogenic diabetes among pediatric patients with positive autoimmunity at diagnosis. Nevertheless, both in our experience [11] and in previously published studies [44,45], the proportion of patients with positive autoimmunity and molecular diagnosis of MODY is the same as expected in the control population, suggesting that autoantibodies are rare in MODY. However, other studies with different approaches show higher prevalence of positive autoantibodies among MODY cases. GADA and IA2A were found in Czech patients with MODY and delayed diabetes onset with insufficient disease control [46]. Pancreatic autoantibodies were also found in a high proportion (17%) in German and Austrian patients with MODY, but this might be an overestimation due to the fact that the diagnosis of MODY was not confirmed by genetic testing in 20% of studied patients. Surprisingly, using the same testing protocol, the positive rate in patients with type 2 diabetes was even greater than in MODY patients [47].

A significantly lower presence of ketoacidosis at diagnosis in autoimmune negative patients was found in our cohort, reflecting a less aggressive onset of diabetes in this subset of patients. Nevertheless, factors involved in DKA development are not yet clear. A recent report has found a correlation between the number of positive antibodies and the severity of ketoacidosis [48], which supports the relation of DKA to the intensity of destruction of pancreatic β-cells. Other reports have found a greater risk of DKA associated with different specific antibodies [48,49]. However, most of the published data find no difference in rates of DKA and presence or absence of autoimmunity [50,51] which substantiates the relationship of DKA to delayed diagnosis rather than to the expression of antigenicity. Our study did not have the capacity to find any other clinical differences among patients with positive and negative autoimmunity and carriers and non-carriers of pathogenic variants, probably due to the small number of patients. The two probands carrying pathogenic variants in this study had been previously diagnosed with T1D, despite the absence of autoimmunity. In these particular cases, neither clinical data nor HLA-DRB1 helped to differentiate MODY. In fact, both patients with MODY had HLA-DRB1 risk alleles for T1D (1 and 2 risk alleles). Furthermore, as the presence of two HLA-DRB1 risk alleles for T1D increases the probability of developing autoimmune diabetes [11] we cannot dismiss the possibility that the proband carrying two HLA-DRB1 risk alleles may also develop autoimmune diabetes in the future.

The main strength of the current study is the well-characterized cohort of T1D patients based on the combination of four autoantibody assays. This enabled us to accurately define the antibody-negative patients who were the target of the molecular testing. Another advantage is the screening approach by NGS that extends the search for genetic variants to twelve genes related to MD as well as the MLPA analysis. The possibility of misdiagnosing patients with rarer forms of monogenic diabetes cannot be completely excluded, as patients may carry variants in other known MD genes not tested in this study or in a gene not yet identified as a monogenic cause of diabetes.

In summary, our study shows that ZnT8A is an autoantibody to be considered for improving the diagnosis of T1D in pediatrics. In addition, at least 8% of pediatric patients suspected of T1D but with undetectable autoimmunity have monogenic diabetes and can benefit from the correct diagnosis of the disease by genetic study.

## Supporting information

S1 Table Characteristics of the population included in the study. (PDF)

## References

[1] Atkinson M, Eisenbarth G, Michels A. Type 1 diabetes. Lancet. 2014; 383(9911):69–82. 10.1016/S0140-6736(13)60591-7 23890997 PMC4380133

[2] Winter W, Schatz D. Autoimmune markers in diabetes. Clin Chem. 2011; 57(2):168–175. 10.1373/clinchem.2010.148205 21127152

[3] Wenzlau J, Juhl K, Yu L, Moua O, Sarkar S, Gottlieb P, et al The cation efflux transporter ZnT8 (Slc30A8) is a major autoantigen in human type 1 diabetes. Proc Natl Acad Sci USA. 2007; 104(43):17040–17045. 10.1073/pnas.0705894104 17942684 PMC2040407

[4] Wang J, Miao D, Babu S, Yu J, Barker J, Klingensmith G, et al Prevalence of autoantibody-negative diabetes is not rare at all ages and increases with older age and obesity. J Clin Endocrinol Metab. 2007; 92(1):88–92. 10.1210/jc.2006-1494 17062766

[5] Erlich H, Valdes A, Noble J, Carlson J, Varney M, Concannon P, et al HLA DR-DQ Haplotypes and Genotypes and Type 1 Diabetes Risk. Diabetes. 2008; 57(4):1084–1092. 10.2337/db07-1331 18252895 PMC4103420

[6] Vaxillaire M, Froguel P. Monogenic diabetes: Implementation of translational genomic research towards precision medicine. J Diabetes. 2016; 8(6):782–795. 10.1111/1753-0407.12446 27390143

[7] Shields BM, Hicks S, Shepherd MH, Colclough K, Hattersley AT, Ellard S. Maturity-onset diabetes of the young (MODY): How many cases are we missing? Diabetologia. 2010; 53(12):2504–2508. 10.1007/s00125-010-1799-4 20499044

[8] Amed S, Oram R. Maturity-Onset Diabetes of the Young (MODY): Making the Right Diagnosis to Optimize Treatment. Can J Diabetes. 2016; 40(5):449–454. 10.1016/j.jcjd.2016.03.002 27130141

[9] Mayer-Davis EJ, Kahkoska AR, Jefferies C, Dabelea D, Balde N, Gong CX, et al ISPAD Clinical Practice Consensus Guidelines 2018: Definition, epidemiology, and classification of diabetes in children and adolescents. Pediatr Diabetes. 2018; 19(S27):7–19. 10.1111/pedi.12773 30226024 PMC7521365

[10] Wolfsdorf JI, Glaser N, Agus M, Fritsch M, Hanas R, Rewers A, et al ISPAD Clinical Practice Consensus Guidelines 2018: Diabetic ketoacidosis and the hyperglycemic hyperosmolar state. Pediatr Diabetes. 2018; 19(S27):155–177. 10.1111/pedi.12701 29900641

[11] Urrutia I, Martínez R, López-Euba T, Velayos T, De La Piscina IM, Bilbao JR, et al Lower frequency of HLA-DRB1 type 1 diabetes risk alleles in pediatric patients with MODY. PLoS One. 2017; 12(1):e0169389 10.1371/journal.pone.0169389 28052112 PMC5214860

[12] Sánchez González E, Carrascosa Lezcano A, Fernández García JM, Ferrández Longás A, López De Lara D, López-Siguero JP. Spanish growth studies: the current situation, their effectiveness and recommendations for their use. An Pediatr. 2011; 74(3):193.e1–e16. 10.1016/j.anpedi.2010.10.005 21237733

[13] Bilbao JR, Rica I, Vazquez JA, Busturia MA, Castano L. Influence of sex and age at onset on autoantibodies against insulin, GAD65 and IA2 in recent onset type 1 diabetic patients. Horm Res. 2000; 54(4):181–185. 10.1159/000053256 11416235

[14] Richards S, Aziz N, Bale S, Bick D, Das S, Gastier-Foster J, et al Standards and guidelines for the interpretation of sequence variants: A joint consensus recommendation of the American College of Medical Genetics and Genomics and the Association for Molecular Pathology. Genet Med. 2015; 17(5):405–424. 10.1038/gim.2015.30 25741868 PMC4544753

[15] Rajan S, Eames SC, Park S-Y, Labno C, Bell GI, Prince VE, et al In vitro processing and secretion of mutant insulin proteins that cause permanent neonatal diabetes. Am J Physiol Endocrinol Metab. 2010; 298(3):E403–E410. 10.1152/ajpendo.00592.2009 19952343 PMC2838531

[16] Støy J, Edghill EL, Flanagan SE, Ye H, Paz VP, Pluzhnikov A, et al Insulin gene mutations as a cause of permanent neonatal diabetes. Proc Natl Acad Sci U S A. 2007; 104(38):15040–15044. 10.1073/pnas.0707291104 17855560 PMC1986609

[17] Brahm AJ, Wang G, Wang J, McIntyre AD, Cao H, Ban MR, et al Genetic Confirmation Rate in Clinically Suspected Maturity-Onset Diabetes of the Young. Can J Diabetes. 2016; 40(6):555–560. 10.1016/j.jcjd.2016.05.010 27634015

[18] Wasserman H, Hufnagel RB, Miraldi Utz V, Zhang K, Valencia CA, Leslie ND, et al Bilateral cataracts in a 6-yr-old with new onset diabetes: a novel presentation of a known INS gene mutation. Pediatr Diabetes. 2016; 17(7):535–539. 10.1111/pedi.12335 26530398 PMC4854816

[19] Bonfanti R, Colombo C, Nocerino V, Massa O, Lampasona V, Iafusco D, et al Insulin gene mutations as cause of diabetes in children negative for five type 1 diabetes autoantibodies. Diabetes Care. 2009; 32(1):123–125. 10.2337/dc08-0783 18840770 PMC2606844

[20] Weber S, Moriniere V, Knüppel T, Charbit M, Dusek J, Ghiggeri GM, et al Prevalence of Mutations in Renal Developmental Genes in Children with Renal Hypodysplasia: Results of the ESCAPE Study. J Am Soc Nephrol. 2006; 17(10):2864–2870. 10.1681/ASN.2006030277 16971658

[21] Karges B, Bergmann C, Scholl K, Heinze E, Rasche FM, Zerres K, et al Digenic inheritance of hepatocyte nuclear factor-1α and -1β with maturity-onset diabetes of the young, polycystic thyroid, and urogenital malformations. Diabetes Care. 2007; 30(6):1613–1614. 10.2337/dc06-2618 17337496

[22] Nicolaou N, Pulit SL, Nijman IJ, Monroe GR, Feitz WFJ, Schreuder MF, et al Prioritization and burden analysis of rare variants in 208 candidate genes suggest they do not play a major role in CAKUT. Kidney Int. 2016; 89(2):476–486. 10.1038/ki.2015.319 26489027

[23] Chèvre J, Hani E, Boutin P, Vaxillaire M, Blanché H, Vionnet N, et al Mutation screening in 18 Caucasian families suggest the existence of other MODY genes. Diabetologia. 1998; 41(9):1017–1023. 10.1007/s001250051025 9754819

[24] Flannick J, Beer NL, Bick AG, Agarwala V, Molnes J, Gupta N, et al Assessing the phenotypic effects in the general population of rare variants in genes for a dominant Mendelian form of diabetes. Nat Genet. 2013; 45(11):1380–1387. 10.1038/ng.2794 24097065 PMC4051627

[25] Colclough K, Bellanné-Chantelot C, Saint-Martin C, Flanagan S, Ellard S. Mutations in the Genes Encoding the Transcription Factors Hepatocyte Nuclear Factor 1 Alpha and 4 Alpha in Maturity-Onset Diabetes of the Young and Hyperinsulinemic Hypoglycemia. Hum Mutat. 2013; 34(5):669–685. 10.1002/humu.22279 23348805

[26] Najmi LA, Aukrust I, Flannick J, Molnes J, Burtt N, Molven A, et al Functional investigations of HNF1A identify rare variants as risk factors for type 2 diabetes in the general population. Diabetes. 2017; 66(2):335–346. 10.2337/db16-0460 27899486 PMC5860263

[27] Chambers C, Fouts A, Dong F, Colclough K, Wang Z, Batish SD, et al Characteristics of maturity onset diabetes of the young in a large diabetes center. Pediatr Diabetes. 2016; 17(5):360–367. 10.1111/pedi.12289 26059258 PMC4934136

[28] Bellanné-Chantelot C, Clauin S, Chauveau D, Collin P, Daumont M, Douillard C, et al Large genomic rearrangements in the hepatocyte nuclear factor-1beta (TCF2) gene are the most frequent cause of maturity-onset diabetes of the young type 5. Diabetes. 2005; 54(11):3126–3132. 10.2337/diabetes.54.11.3126 16249435

[29] Dopazo J, Amadoz A, Bleda M, Garcia-Alonso L, Alemán A, García-García F, et al 267 Spanish Exomes Reveal Population-Specific Differences in Disease-Related Genetic Variation. Mol Biol Evol. 2016; 33(5):1205–1218. 10.1093/molbev/msw005 26764160 PMC4839216

[30] Madariaga L, Morinière V, Jeanpierre C, Bouvier R, Loget P, Martinovic J, et al Severe prenatal renal anomalies associated with mutations in HNF1B or PAX2 genes. Clin J Am Soc Nephrol. 2013; 8(7):1179–1187. 10.2215/CJN.10221012 23539225 PMC3700697

[31] Faguer S, Decramer S, Chassaing N, Bellanné-Chantelot C, Calvas P, Beaufils S, et al Diagnosis, management, and prognosis of HNF1B nephropathy in adulthood. Kidney Int. 2011; 80(7):768–776. 10.1038/ki.2011.225 21775974

[32] Andersson C, Kolmodin M, Ivarsson S, Carlsson A, Forsander G, Lindblad B, et al Islet cell antibodies (ICA) identify autoimmunity in children with new onset diabetes mellitus negative for other islet cell antibodies. Pediatr Diabetes. 2014; 15(5):336–344. 10.1111/pedi.12093 24206368

[33] Verkauskiene R, Danyte E, Dobrovolskiene R, Stankute I, Simoniene D, Razanskaite-Virbickiene D, et al The course of diabetes in children, adolescents and young adults: Does the autoimmunity status matter? BMC Endocr Disord. 2016; 16(1):61 10.1186/s12902-016-0145-3 27842589 PMC5109672

[34] Johansson BB, Irgens HU, Molnes J, Sztromwasser P, Aukrust I, Juliusson PB, et al Targeted next-generation sequencing reveals MODY in up to 6.5% of antibody-negative diabetes cases listed in the Norwegian Childhood Diabetes Registry. Diabetologia. 2017; 60(4):625–635. 10.1007/s00125-016-4167-1 27913849

[35] Gandica RG, Chung WK, Deng L, Goland R, Gallagher MP. Identifying monogenic diabetes in a pediatric cohort with presumed type 1 diabetes. Pediatr Diabetes. 2015; 16(3):227–233. 10.1111/pedi.12150 25082184 PMC4767163

[36] Rubio-Cabezas O, Edghill EL, Argente J, Hattersley AT. Testing for monogenic diabetes among children and adolescents with antibody-negative clinically defined Type 1 diabetes. Diabet Med. 2009; 26(10):1070–1074. 10.1111/j.1464-5491.2009.02812.x 19900242

[37] Ushijima K, Fukami M, Ayabe T, Narumi S, Okuno M, Nakamura A, et al Comprehensive screening for monogenic diabetes in 89 Japanese children with insulin-requiring antibody-negative type 1 diabetes. Pediatr Diabetes. 2018; 19(2):243–250. 10.1111/pedi.12544 28597946

[38] Leighton E, Sainsbury CA, Jones GC. A Practical Review of C-Peptide Testing in Diabetes. Diabetes Ther. 2017; 8(3):475–487. 10.1007/s13300-017-0265-4 28484968 PMC5446389

[39] Reinehr T. Type 2 diabetes mellitus in children and adolescents. World J Diabetes. 2013; 4(6):270–281. 10.4239/wjd.v4.i6.270 24379917 PMC3874486

[40] Aguayo A, Vela A, Aniel-Quiroga A, Blarduni E, Fernández C, Grau G, et al Absence of diabetes mellitus type 2 in obese children and adolescents in the north of Spain. J Pediatr Endocrinol Metab. 2013; 26(1–2):25–29. 10.1515/jpem-2012-0200 23329743

[41] Massa O, Alessio M, Russo L, Nardo G, Bonetto V, Bertuzzi F, et al Serological Proteome Analysis (SERPA) as a tool for the identification of new candidate autoantigens in type 1 diabetes. J Proteomics. 2013; 82:263–273. 10.1016/j.jprot.2013.02.030 23500132

[42] Patel KA, Weedon MN, Shields BM, Pearson ER, Hattersley AT, McDonald TJ. Zinc Transporter 8 Autoantibodies (ZnT8A) and a Type 1 Diabetes Genetic Risk Score Can Exclude Individuals With Type 1 Diabetes From Inappropriate Genetic Testing for Monogenic Diabetes. Diabetes Care. 2018; 42(2):e16–e17. 10.2337/dc18-0373 30409810 PMC6984957

[43] Heneberg P, Šimčíková D, Čecháková M, Rypáčková B, Kučera P, Anděl M. Autoantibodies against ZnT8 are rare in Central-European LADA patients and absent in MODY patients, including those positive for other autoantibodies. J Diabetes Complications. 2019; 33(1):46–52. 10.1016/j.jdiacomp.2018.10.004 30377089

[44] McDonald T, Colclough K, Brown R, Shields B, Shepherd M, Bingley P, et al Islet autoantibodies can discriminate maturity-onset diabetes of the young (MODY) from Type1 diabetes. Diabet Med. 2011; 28(9):1028–1033. 10.1111/j.1464-5491.2011.03287.x 21395678

[45] Johnson SR, Ellis JJ, Leo PJ, Anderson LK, Ganti U, Harris JE, et al Comprehensive genetic screening: The prevalence of maturity-onset diabetes of the young gene variants in a population-based childhood diabetes cohort. Pediatr Diabetes. 2019; 20(1):57–64. 10.1111/pedi.12766 30191644

[46] Urbanová J, Rypáčková B, Procházková Z, Kučera P, Černá M, Anděl M, et al Positivity for islet cell autoantibodies in patients with monogenic diabetes is associated with later diabetes onset and higher HbA1c level. Diabet Med. 2014; 31(4):466–471. 10.1111/dme.12314 24102923

[47] Schober E, Rami B, Grabert M, Thon A, Kapellen T, Reinehr T, et al Phenotypical aspects of maturity-onset diabetes of the young (MODY diabetes) in comparison with Type 2 diabetes mellitus (T2DM) in children and adolescents: Experience from a large multicentre database. Diabet Med. 2009; 26(5):466–473. 10.1111/j.1464-5491.2009.02720.x 19646184

[48] Niechciał E, Rogowicz-Frontczak A, Piłaciński S, Fichna M, Skowrońska B, Fichna P, et al Autoantibodies against zinc transporter 8 are related to age and metabolic state in patients with newly diagnosed autoimmune diabetes. Acta Diabetol. 2018; 55(3):287–294. 10.1007/s00592-017-1091-x 29327148 PMC5829102

[49] Vicinanza A, Messaaoui A, Tenoutasse S, Dorchy H. Diabetic ketoacidosis in children newly diagnosed with type 1 diabetes mellitus: role of demographic, clinical and biochemical features along with genetic and immunological markers as risk factors. A twenty-year experience in a tertiary Belgian centre. Pediatr Diabetes. Forthcoming 2019 10.1111/pedi.12864 31038262

[50] Bravis V, Kaur A, Walkey HC, Godsland IF, Misra S, Bingley PJ, et al Relationship between islet autoantibody status and the clinical characteristics of children and adults with incident type 1 diabetes in a UK cohort. BMJ Open. 2018; 8(4):e020904 10.1136/bmjopen-2017-020904 29622578 PMC5893930

[51] Östman J, Landin-Olsson M, Törn C, Palmer J, Lernmark A, Arnqvist H, et al Ketoacidosis in young adults is not related to the islet antibodies at the diagnosis of Type 1 diabetes mellitus-a nationwide study. Diabet Med. 2000; 17(4):269–274. 10.1046/j.1464-5491.2000.00265.x 10821292

