# Supplementary material for: Negative autoimmunity in a Spanish pediatric cohort suspected of type 1 diabetes, could it be monogenic diabetes?
Source: PLoS One. 2019 Jul 31;14(7):e0220634. doi: 10.1371/journal.pone.0220634 (PMC6668821; doi:10.1371/journal.pone.0220634)
Supplement: S1 Table — (PDF) [file pone.0220634.s001.pdf]

Patient ID	Gender (Female:F; Male:M)	Age at onset (years)	DKA	IAA	GADA	IA2A	ZnT8A	First degree relatives with DM	Parents with DM	Siblings with DM	DRB1_ Allele1	DRB1_ Allele2
1	F	9.3	NO	NEG	NEG	NEG	NEG	NO	NO	NO	0301	0301
2	M	14.7	NO	NEG	NEG	NEG	NEG	NO	NO	NO	0102	1101
3	F	14.3	NO	NEG	NEG	NEG	NEG	YES	MOTHER	NO	0404	0802
4	M	7.0	YES	NEG	NEG	NEG	NEG	NO	NO	NO	0403	1102
5	M	13.3	NO	NEG	NEG	NEG	NEG	NO	NO	NO	0301	1601
6	M	16.0	NO	NEG	NEG	NEG	NEG	NO	NO	NO	0301	0301
7	M	8.1	YES	NEG	NEG	NEG	NEG	NO	NO	NO	0301	1601
8	M	15.9	NO	NEG	NEG	NEG	NEG	NO	NO	NO	0404	0901
9	F	9.3	NO	NEG	NEG	NEG	NEG	NO	NO	NO	0301	0405
10	F	10.8	NO	NEG	NEG	NEG	NEG	NO	NO	NO	0301	0403
11	M	5.7	NO	NEG	NEG	NEG	NEG	NO DATA	NO DATA	NO DATA	0405	1401
12	M	7.3	NO	NEG	NEG	NEG	NEG	NO	NO	NO	0301	0701
13	M	8.0	NO	NEG	NEG	NEG	NEG	NO	NO	NO	0301	0801
14	F	13.8	YES	NEG	NEG	NEG	NEG	NO	NO	NO	0101	0401
15	M	7.4	NO	NEG	NEG	NEG	NEG	NO	NO	NO	0401	0701
16	M	9.9	NO	NEG	NEG	NEG	NEG	NO	NO	NO	03	04
17	F	8.5	NO	NEG	NEG	NEG	NEG	NO	NO	NO	0301	0405
18	M	12.0	NO	NEG	NEG	NEG	NEG	YES	MOTHER	YES	0301	0402
19	M	14.4	NO	NEG	NEG	NEG	NEG	NO	NO	NO	0301	0301
20	M	0.8	YES	NEG	NEG	NEG	NEG	YES	MOTHER	NO	0301	0404
21	F	2.2	NO DATA	NEG	NEG	NEG	NEG	NO	NO	NO	0301	0401
22	M	5.6	NO	NEG	NEG	NEG	NEG	NO	NO	NO	0301	0401
23	F	6.3	NO	NEG	NEG	NEG	NEG	NO	NO	NO	0301	0404
24	M	1.5	NO	POS	POS	POS	POS	YES	MOTHER	NO	0405	0901
25	M	11.9	NO	NEG	NEG	POS	POS	YES	FATHER	NO	0404	0405
26	F	2.3	NO	POS	POS	POS	NEG	NO	NO	NO	0408	1601
27	M	12.5	NO	POS	POS	NEG	NEG	YES	FATHER	NO	0301	0301
28	F	15.0	NO	POS	POS	POS	POS	NO	NO	NO	0102	0301
29	F	10.5	YES	POS	POS	POS	NEG	YES	MOTHER	NO	0401	0401
30	F	9.7	NO	POS	POS	POS	POS	NO	NO	NO	0405	0701
31	F	12.8	YES	POS	POS	NEG	POS	NO	NO	NO	0101	0301
32	M	3.3	YES	POS	POS	POS	POS	NO	NO	NO	0401	0405
33	M	2.8	NO	POS	NEG	POS	NEG	YES	FATHER	NO	0404	0405
34	F	12.7	YES	NEG	POS	POS	POS	YES	NO	YES	0401	1302
35	F	3.7	NO	POS	NEG	POS	POS	YES	FATHER	NO	0301	0404
36	F	6.0	NO	POS	POS	POS	POS	NO	NO	NO	0301	0403
37	M	14.4	NO	NEG	POS	POS	POS	NO	NO	NO	0404	1302
38	M	5.5	NO	POS	POS	POS	NEG	NO	NO	NO	0405	1101
39	F	8.0	YES	POS	POS	POS	NEG	NO	NO	NO	0301	1302
40	M	11.9	YES	NEG	POS	NEG	NEG	YES	MOTHER	NO	0301	1301
41	M	11.2	NO	NEG	POS	NEG	POS	NO	NO	NO	0101	0301
42	M	15.3	NO	POS	POS	POS	POS	NO	NO	NO	0402	0701
43	M	13.9	YES	NEG	NEG	POS	NEG	NO	NO	NO	0301	0401
44	F	3.2	YES	POS	POS	POS	NEG	NO	NO	NO	0301	0405
45	F	6.4	YES	POS	POS	NEG	NEG	NO	NO	NO	0301	1501
46	M	12.0	NO	NEG	NEG	POS	POS	YES	MOTHER	NO	0301	0401
47	F	12.9	NO	POS	POS	POS	POS	NO	NO	NO	0301	0405
48	F	3.7	NO	POS	POS	POS	POS	YES	NO	YES	0301	0301
49	F	5.2	YES	POS	POS	POS	POS	YES	NO	YES	0301	0901
50	M	7.4	NO	POS	POS	POS	NEG	NO	NO	NO	0301	0402

Patient ID	Gender (Female:F; Male:M)	Age at onset (years)	DKA	IAA	GADA	IA2A	ZnT8A	First degree relatives with DM	Parents with DM	Siblings with DM	DRB1_ Allele1	DRB1_ Allele2
51	F	7.2	YES	POS	POS	POS	NEG	NO	NO	NO	0401	1301
52	F	6.3	NO	POS	POS	POS	POS	NO	NO	NO	0301	0402
53	F	2.5	YES	POS	POS	NEG	NEG	NO	NO	NO	0301	0402
54	F	9.3	YES	POS	POS	NEG	POS	NO	NO	NO	0405	0701
55	M	11.4	NO	POS	POS	POS	POS	NO	NO	NO	0404	1601
56	M	4.3	NO	POS	NEG	NEG	NEG	NO	NO	NO	0103	0301
57	M	8.5	NO	POS	POS	POS	POS	NO DATA	NO DATA	NO DATA	0301	0401
58	F	12.6	YES	POS	POS	POS	POS	NO	NO	NO	0101	1302
59	F	5.1	YES	POS	POS	NEG	POS	NO	NO	NO	0401	1301
60	M	14.8	NO	POS	POS	POS	NEG	NO	NO	NO	0301	0405
61	M	8.7	YES	NEG	POS	NEG	NEG	NO	NO	NO	0101	0301
62	F	4.8	NO	NEG	NEG	POS	NEG	YES	MOTHER	NO	0103	0405
63	F	6.5	YES	POS	POS	POS	NEG	NO	NO	NO	0301	0401
64	M	2.8	NO	POS	POS	NEG	NEG	NO	NO	NO	0301	1602
65	M	12.7	YES	NEG	POS	NEG	POS	NO	NO	NO	0102	1302
66	M	11.1	YES	NEG	POS	NEG	NEG	NO	NO	NO	0102	0301
67	M	1.4	NO	POS	POS	POS	NEG	YES	FATHER	NO	0301	0301
68	M	12.1	YES	POS	POS	NEG	NEG	NO	NO	NO	0301	1601
69	F	4.4	NO	POS	POS	POS	NEG	NO	NO	NO	0301	0701
70	F	3.7	NO	POS	NEG	NEG	NEG	YES	MOTHER	YES	0301	0404
71	F	8.7	YES	NEG	POS	POS	NEG	NO	NO	NO	0301	0801
72	M	11.1	YES	POS	POS	POS	POS	YES	FATHER	NO	0301	0301
73	M	4.3	NO	NEG	POS	NEG	NEG	NO	NO	NO	0301	0301
74	M	8.3	NO	POS	POS	POS	POS	NO	NO	NO	0301	1406
75	F	9.9	NO	POS	POS	POS	NEG	NO	NO	NO	0101	0301
76	M	7.1	NO	POS	POS	NEG	NEG	NO	NO	NO	0102	0701
77	F	3.6	NO	POS	POS	POS	NEG	NO DATA	NO DATA	NO DATA	0102	1501
78	F	12.4	NO	POS	NEG	POS	POS	NO	NO	NO	0301	0701
79	F	8.0	NO	POS	POS	POS	POS	NO	NO	NO	0301	0405
80	M	12.0	NO	NEG	POS	POS	POS	NO	NO	NO	0301	1301
81	M	13.7	YES	POS	POS	POS	NEG	NO	NO	NO	0301	0701
82	F	1.9	NO	NEG	POS	NEG	NEG	NO	NO	NO	0301	0301
83	F	7.8	YES	POS	NEG	POS	NEG	NO	NO	NO	0101	0401
84	F	11.3	NO	POS	POS	POS	POS	NO	NO	NO	0301	0901
85	F	2.1	YES	POS	POS	NEG	NEG	NO	NO	NO	0301	0301
86	F	2.3	YES	POS	POS	NEG	NEG	NO	NO	NO	1301	1308
87	M	2.7	NO	POS	POS	NEG	POS	YES	FATHER	NO	0301	0301
88	F	1.0	NO	POS	POS	NEG	NEG	NO	NO	NO	0301	0405
89	M	9.9	YES	POS	POS	POS	POS	NO	NO	NO	0402	0801
90	F	6.8	NO	POS	POS	POS	NEG	NO	NO	NO	0402	0701
91	M	3.3	NO	POS	POS	POS	NEG	NO	NO	NO	0301	0301
92	M	13.6	YES	NEG	NEG	NEG	POS	NO	NO	NO	0401	0701
93	F	6.3	NO	POS	POS	POS	NEG	NO	NO	NO	0301	0402
94	F	7.4	NO	POS	POS	POS	POS	NO	NO	NO	0901	1302
95	M	4.8	NO	POS	POS	POS	NEG	NO	NO	NO	0301	0402
96	F	10.4	NO	NEG	POS	NEG	NEG	NO	NO	NO	0102	0701
97	F	2.1	YES	POS	POS	NEG	NEG	NO	NO	NO	0404	0301
98	M	12.8	NO	NEG	POS	POS	POS	NO	NO	NO	0301	0404
99	M	12.8	NO	POS	POS	POS	POS	NO	NO	NO	0301	1201
100	F	10.0	NO	POS	NEG	POS	NEG	NO	NO	NO	0101	0402

Patient ID	Gender (Female:F; Male:M)	Age at onset (years)	DKA	IAA	GADA	IA2A	ZnT8A	First degree relatives with DM	Parents with DM	Siblings with DM	DRB1_ Allele1	DRB1_ Allele2
101	F	8.4	YES	NEG	POS	POS	POS	NO	NO	NO	0405	0301
102	M	13.0	YES	POS	POS	POS	POS	NO	NO	NO	0402	1102
103	F	13.5	NO	POS	POS	POS	POS	NO	NO	NO	0101	0301
104	F	10.8	NO	POS	POS	POS	POS	NO	NO	NO	0301	0404
105	F	6.8	YES	POS	POS	POS	POS	NO	NO	NO	0301	0401
106	M	13.4	YES	POS	POS	POS	NEG	NO	NO	NO	0301	0701
107	M	11.2	YES	NEG	POS	POS	NEG	NO	NO	NO	0301	0405
108	F	1.4	NO	POS	POS	POS	NEG	NO	NO	NO	0401	0701
109	M	3.8	NO	NEG	POS	POS	POS	NO	NO	NO	0301	0405
110	F	9.5	YES	NEG	POS	POS	POS	NO	NO	NO	0301	0401
111	F	13.3	NO	POS	POS	POS	POS	NO	NO	NO	0301	0402
112	F	8.5	NO	POS	POS	POS	POS	YES	BOTH	NO	0101	0404
113	M	10.7	NO	NEG	POS	POS	NEG	YES	FATHER	NO	0301	0404
114	F	11.6	NO	NEG	POS	POS	POS	NO	NO	NO	0301	0301
115	M	13.3	NO	POS	POS	POS	POS	NO	NO	NO	0301	0301
116	F	11.9	NO	NEG	POS	NEG	NEG	NO	NO	NO	0101	0301
117	M	15.5	NO	POS	POS	POS	POS	NO	NO	NO	0301	0401
118	F	13.7	YES	NEG	POS	NEG	POS	YES	NO	YES	0101	0301
119	F	10.3	NO	NEG	POS	NEG	POS	NO	NO	NO	0301	1301
120	F	13.7	YES	NEG	POS	POS	POS	NO	NO	NO	0301	0405
121	M	14.3	YES	NEG	POS	NEG	POS	NO	NO	NO	0101	0101
122	F	3.8	NO	POS	POS	NEG	NEG	YES	FATHER	NO	0301	0401
123	M	0.8	YES	POS	NEG	NEG	NEG	NO	NO	NO	0301	0301
124	F	7.8	NO	POS	POS	POS	POS	NO	NO	NO	0301	0301
125	F	13.6	YES	NEG	NEG	POS	POS	NO	NO	NO	0401	0405
126	F	10.4	YES	POS	NEG	NEG	NEG	NO	NO	NO	0701	0801
127	F	5.4	NO	POS	NEG	POS	NEG	NO	NO	NO	0301	0401
128	M	10.3	NO	NEG	POS	POS	POS	NO	NO	NO	0401	1301
129	M	8.7	NO	POS	NEG	POS	NEG	YES	FATHER	NO	0301	0405
130	M	12.5	YES	NEG	POS	POS	POS	NO	NO	NO	0101	0301
131	M	13.4	NO	POS	POS	POS	POS	NO	NO	NO	0404	1301
132	M	11.6	NO	NEG	POS	POS	POS	NO	NO	NO	0405	1301
133	F	12.2	NO	POS	POS	POS	POS	NO	NO	NO	0301	1404
134	M	3.1	YES	POS	NEG	POS	POS	NO	NO	NO	0301	0402
135	M	5.3	NO	POS	POS	NEG	POS	NO	NO	NO	0301	0301
136	M	7.1	NO	NEG	POS	NEG	NEG	NO	NO	NO	0301	0801
137	F	6.5	NO	POS	POS	POS	POS	NO	NO	NO	0401	0801
138	M	7.6	NO	POS	POS	POS	NEG	YES	FATHER	NO	0301	0401
139	F	11.3	NO	NEG	POS	POS	POS	YES	NO	YES	0301	1104
140	F	11.5	YES	NEG	NEG	POS	POS	YES	FATHER	NO	0405	0801
141	F	10.5	NO	POS	POS	NEG	POS	NO	NO	NO	0401	0405
142	M	12.5	NO	NEG	POS	POS	POS	NO	NO	NO	0301	0404
143	F	2.0	YES	POS	POS	POS	NEG	NO	NO	NO	0101	0405
144	F	8.3	YES	NEG	NEG	POS	POS	NO	NO	NO	0301	1302
145	F	8.4	YES	POS	POS	POS	POS	NO	NO	NO	0405	0701
146	F	5.1	NO	NEG	POS	POS	NEG	NO	NO	NO	0301	0301
147	F	9.1	NO	POS	POS	POS	POS	NO	NO	NO	0301	0301
148	M	12.8	YES	POS	POS	NEG	POS	YES	FATHER	NO	0301	0401
149	M	11.0	YES	POS	POS	POS	POS	NO DATA	NO DATA	NO DATA	0301	0701
150	M	12.0	NO	NEG	POS	NEG	NEG	YES	FATHER	YES	0301	0301

Patient ID	Gender (Female:F; Male:M)	Age at onset (years)	DKA	IAA	GADA	IA2A	ZnT8A	First degree relatives with DM	Parents with DM	Siblings with DM	DRB1_ Allele1	DRB1_ Allele2
151	F	6.1	YES	NEG	NEG	POS	POS	NO	NO	NO	0301	1501
152	F	11.8	NO	NEG	POS	NEG	POS	NO	NO	NO	0301	1601
153	F	8.8	YES	NEG	NEG	POS	NEG	NO	NO	NO	0301	0401
154	M	8.2	NO	NEG	POS	POS	POS	NO	NO	NO	0401	0405
155	M	7.5	NO	NEG	POS	NEG	NEG	NO	NO	NO	0101	0403
156	F	14.3	NO	POS	POS	NEG	POS	NO	NO	NO	0301	0301
157	M	5.5	NO	NEG	POS	NEG	POS	NO	NO	NO	0301	0301
158	F	0.8	NO	POS	POS	POS	NEG	NO	NO	NO	0301	0701
159	M	8.3	NO	POS	POS	POS	NEG	YES	MOTHER	NO	0301	0701
160	F	3.4	NO	NEG	POS	POS	POS	NO	NO	NO	0301	0701
161	F	12.9	YES	POS	POS	POS	POS	NO	NO	NO	0301	0401
162	F	5.7	YES	POS	POS	NEG	NEG	NO	NO	NO	0301	0402
163	M	7.7	YES	POS	NEG	POS	NEG	NO	NO	NO	0402	1302
164	F	7.2	NO	POS	NEG	POS	NEG	NO	NO	NO	0405	0405
165	F	10.6	YES	POS	POS	NEG	POS	YES	FATHER	NO	0102	0801
166	M	2.3	YES	POS	NEG	POS	NEG	NO	NO	NO	0301	0402
167	F	7.3	YES	NEG	POS	POS	POS	NO	NO	NO	0101	0407
168	M	1.9	NO	POS	POS	POS	POS	YES	MOTHER	NO	0301	0403
169	M	8.3	NO	POS	POS	POS	POS	NO	NO	NO	0402	0405
170	M	6.8	YES	POS	NEG	NEG	NEG	NO	NO	NO	0701	0801
171	F	15.0	YES	POS	POS	POS	POS	NO	NO	NO	0301	0405
172	M	7.8	NO	POS	POS	POS	NEG	NO	NO	NO	0301	1302
173	M	13.1	NO	NEG	POS	NEG	NEG	NO	NO	NO	0301	0301
174	F	9.6	NO	POS	POS	POS	POS	NO	NO	NO	0101	0401
175	M	5.7	NO	POS	NEG	POS	NEG	NO	NO	NO	0301	0405
176	F	11.3	NO	NEG	POS	POS	POS	NO	NO	NO	0301	0301
177	F	11.6	YES	NEG	POS	POS	POS	NO	NO	NO	0301	0301
178	F	11.1	YES	NEG	POS	POS	POS	NO	NO	NO	0301	0404
179	M	5.6	NO	POS	NEG	POS	POS	NO	NO	NO	0402	0701
180	M	15.4	YES	POS	POS	POS	POS	NO	NO	NO	0301	0401
181	F	10.0	NO	POS	POS	POS	POS	NO	NO	NO	1201	1301
182	F	7.7	NO	POS	POS	POS	NEG	YES	FATHER	YES	0401	1104
183	M	12.5	NO	NEG	NEG	NEG	POS	NO	NO	NO	0301	1303
184	M	4.5	NO	POS	NEG	POS	NEG	NO	NO	NO	0101	0405
185	M	11.8	YES	POS	POS	POS	POS	NO	NO	NO	0301	0401
186	M	9.2	NO	NEG	POS	NEG	NEG	NO	NO	NO	0301	0301
187	M	5.2	YES	POS	NEG	POS	POS	NO	NO	NO	0401	1303
188	F	7.7	NO	POS	POS	NEG	NEG	NO	NO	NO	0301	1601
189	F	13.4	NO	NEG	NEG	POS	NEG	YES	NO	YES	0102	0405
190	F	11.0	NO	POS	POS	POS	POS	NO	NO	NO	0301	0301
191	F	2.4	NO	POS	POS	POS	NEG	NO	NO	NO	0301	0405
192	M	1.2	NO	POS	POS	NEG	POS	NO	NO	NO	0301	0405
193	F	10.4	YES	POS	POS	NEG	POS	NO	NO	NO	0301	0402
194	M	3.3	YES	NEG	POS	POS	POS	NO	NO	NO	0301	0401
195	F	13.5	YES	NEG	POS	NEG	NEG	NO	NO	NO	0101	0406
196	F	11.8	NO	POS	POS	POS	POS	NO	NO	NO	0301	0402
197	F	14.8	NO	NEG	POS	POS	POS	NO	NO	NO	0101	0102
198	M	4.0	NO	POS	POS	POS	NEG	NO	NO	NO	0301	0401
199	M	9.1	YES	POS	POS	POS	POS	NO	NO	NO	0301	0402
200	F	6.5	NO	POS	NEG	POS	NEG	NO	NO	NO	0301	0404

Patient ID	Gender (Female:F; Male:M)	Age at onset (years)	DKA	IAA	GADA	IA2A	ZnT8A	First degree relatives with DM	Parents with DM	Siblings with DM	DRB1_ Allele1	DRB1_ Allele2
201	M	11.8	YES	NEG	POS	POS	POS	NO	NO	NO	0301	1101
202	F	10.4	NO	POS	POS	POS	POS	YES	MOTHER	NO	0301	0404
203	M	14.8	NO	NEG	POS	POS	POS	NO	NO	NO	0301	1302
204	M	12.8	NO	NEG	POS	POS	POS	YES	NO	YES	0301	0404
205	F	11.8	NO	POS	POS	POS	POS	NO	NO	NO	0301	0301
206	M	6.5	YES	POS	POS	POS	POS	NO	NO	NO	0301	0404
207	M	6.2	NO	NEG	NEG	POS	POS	NO	NO	NO	0401	0405
208	M	12.2	NO	POS	POS	POS	NEG	NO	NO	NO	0301	0301
209	F	9.8	NO	NEG	POS	POS	NEG	NO	NO	NO	0101	0301
210	M	14.3	YES	NEG	POS	POS	POS	NO	NO	NO	0301	1001
211	F	4.8	NO	POS	POS	POS	NEG	NO	NO	NO	0301	0401
212	F	4.0	YES	POS	POS	POS	POS	NO	NO	NO	0901	1302
213	F	0.8	YES	POS	POS	NEG	NEG	NO DATA	NO DATA	NO DATA	0301	0402
214	F	11.0	NO	POS	POS	POS	POS	NO	NO	NO	0301	0404
215	F	9.9	NO	POS	POS	POS	NEG	NO	NO	NO	0301	0401
216	M	14.9	NO	POS	NEG	POS	POS	NO	NO	NO	1302	1303
217	M	12.3	YES	POS	POS	POS	POS	NO	NO	NO	0301	0405
218	F	9.9	YES	POS	POS	POS	POS	NO	NO	NO	0301	0405
219	F	11.4	NO	NEG	POS	NEG	NEG	NO	NO	NO	0101	0405
220	M	10.4	NO	NEG	POS	POS	POS	NO	NO	NO	0301	0403
221	M	10.7	YES	POS	POS	NEG	POS	YES	FATHER	NO	0301	1301
222	M	12.9	YES	POS	NEG	NEG	NEG	NO	NO	NO	0301	0301
223	F	16.8	NO	POS	POS	POS	POS	NO	NO	NO	0401	0101
224	M	8.8	NO	POS	POS	POS	NEG	NO	NO	NO	0301	0301
225	M	11.3	YES	POS	NEG	POS	POS	NO	NO	NO	0402	0405
226	F	7.4	YES	POS	POS	NEG	POS	YES	NO	YES	0301	0404
227	M	8.4	YES	POS	POS	NEG	NEG	NO	NO	NO	03	03
228	F	10.5	NO	POS	POS	POS	POS	YES	MOTHER	NO	0102	0701
229	M	14.9	NO	POS	POS	POS	POS	YES	NO	YES	0301	0301
230	F	2.1	NO	POS	NEG	POS	NEG	YES	MOTHER	NO	0301	0402
231	F	3.0	NO	POS	NEG	POS	POS	NO	NO	NO	0402	0405
232	F	8.0	YES	NEG	NEG	POS	POS	NO	NO	NO	0102	0407
233	F	8.3	NO	NEG	POS	POS	POS	NO	NO	NO	0404	0701
234	F	6.5	NO	POS	NEG	POS	POS	NO	NO	NO	0301	0402
235	M	9.8	NO	NEG	NEG	NEG	POS	NO	NO	NO	0101	0301
236	F	11.3	YES	NEG	NEG	POS	POS	NO	NO	NO	0405	1302
237	F	13.4	YES	POS	POS	NEG	POS	NO	NO	NO	0301	1302
238	M	0.8	YES	POS	NEG	NEG	NEG	YES	MOTHER	NO	01	03
239	F	12.5	YES	POS	POS	POS	POS	NO	NO	NO	0102	0402
240	M	4.3	NO	POS	POS	NEG	POS	NO	NO	NO	0101	0301
241	M	8.3	NO	POS	NEG	POS	NEG	NO	NO	NO	0401	0404
242	F	6.3	NO	POS	POS	POS	POS	NO	NO	NO	0402	1303
243	F	6.0	NO	POS	POS	POS	NEG	NO	NO	NO	07	03
244	F	10.0	YES	POS	POS	POS	POS	NO	NO	NO	0301	0701
245	F	8.3	NO	POS	POS	POS	NEG	NO	NO	NO	0301	0404
246	F	8.4	NO	POS	POS	POS	POS	NO	NO	NO	0405	1302
247	M	5.4	YES	POS	NEG	POS	POS	NO	NO	NO	0101	0404
248	F	5.2	NO	POS	NEG	POS	POS	YES	FATHER	NO	0402	0701
249	M	11.0	YES	NEG	POS	POS	POS	NO	NO	NO	0301	0404
250	M	8.8	NO	NEG	POS	POS	POS	NO	NO	NO	0301	0402

Patient ID	Gender (Female:F; Male:M)	Age at onset (years)	DKA	IAA	GADA	IA2A	ZnT8A	First degree relatives with DM	Parents with DM	Siblings with DM	DRB1_ Allele1	DRB1_ Allele2
251	M	13.7	NO	POS	POS	POS	POS	NO	NO	NO	0301	0404
252	F	9.2	YES	POS	NEG	POS	POS	NO	NO	NO	0101	0301
253	F	11.4	YES	POS	POS	NEG	NEG	NO	NO	NO	0301	0401
254	M	3.5	YES	POS	POS	POS	NEG	NO	NO	NO	0301	0403
255	M	11.3	NO	POS	POS	POS	POS	NO	NO	NO	0102	0402
256	M	14.6	YES	POS	POS	NEG	POS	NO	NO	NO	0401	0404
257	F	14.9	YES	POS	POS	POS	NEG	NO	NO	NO	0301	0405
258	F	10.5	NO	POS	POS	POS	POS	NO	NO	NO	0405	0701
259	M	9.6	NO	NEG	POS	NEG	POS	YES	NO	YES	0301	1302
260	M	15.2	NO	NEG	POS	NEG	POS	NO	NO	NO	0102	0103
261	F	8.9	NO	POS	POS	POS	POS	NO	NO	NO	0301	0402
262	M	13.8	NO	POS	POS	POS	POS	NO	NO	NO	0301	0405
263	F	2.9	YES	POS	NEG	POS	POS	NO	NO	NO	0301	0402
264	M	10.3	NO	NEG	POS	NEG	NEG	NO	NO	NO	0301	0701
265	M	13.8	NO	NEG	POS	POS	POS	NO	NO	NO	0404	0405
266	M	14.4	YES	POS	POS	POS	NEG	NO	NO	NO	07	03
267	F	7.8	NO	NEG	POS	NEG	POS	YES	FATHER	NO	0301	0405
268	M	3.6	NO	POS	NEG	POS	NEG	NO	NO	NO	0405	0405
269	M	3.3	NO	POS	NEG	POS	POS	NO	NO	NO	0405	1302
270	M	1.1	YES	POS	POS	POS	NEG	NO	NO	NO	0301	0301
271	M	6.0	NO	POS	NEG	POS	NEG	NO	NO	NO	0102	0301
272	F	13.0	NO	POS	POS	POS	POS	NO	NO	NO	0301	0405
273	M	12.8	YES	POS	POS	POS	POS	NO	NO	NO	0701	0701
274	M	12.3	NO	POS	POS	NEG	POS	NO	NO	NO	0701	0701
275	M	8.8	NO	NEG	POS	NEG	NEG	NO	NO	NO	0301	0404
276	M	2.9	YES	POS	POS	POS	POS	NO	NO	NO	0301	0402
277	M	6.0	YES	POS	POS	POS	POS	NO	NO	NO	0408	0701
278	M	13.6	NO	NEG	POS	POS	POS	NO	NO	NO	0404	0404
279	F	11.7	NO	NEG	POS	POS	POS	NO	NO	NO	0301	0301
280	M	12.6	YES	POS	POS	POS	POS	NO	NO	NO	0405	1101
281	M	11.2	NO	NEG	POS	POS	NEG	YES	FATHER	NO	0301	0301
282	M	14.7	NO	NEG	POS	NEG	NEG	NO	NO	NO	0301	0301
283	F	7.3	YES	POS	POS	POS	NEG	NO	NO	NO	0301	1001
284	M	11.5	NO	POS	POS	NEG	POS	NO	NO	NO	0301	0401
285	M	8.6	NO	POS	POS	NEG	POS	NO	NO	NO	0401	0405
286	M	6.4	NO	POS	NEG	POS	POS	NO	NO	NO	0301	0405
287	M	8.3	YES	POS	NEG	POS	NEG	NO	NO	NO	0402	0404
288	M	11.5	YES	NEG	NEG	POS	NEG	NO	NO	NO	0301	0701
289	F	12.1	YES	POS	POS	POS	POS	YES	ATHER&MOTHE	NO	0102	0402
290	F	9.3	NO	POS	POS	POS	POS	NO	NO	NO	0301	0405
291	F	8.6	YES	POS	POS	POS	NEG	NO	NO	NO	0403	0410
292	M	3.8	YES	POS	NEG	POS	NEG	YES	MOTHER	NO	0301	0405
293	M	12.3	NO	NEG	POS	NEG	POS	YES	MOTHER	NO	0101	0301
294	M	7.4	NO	POS	NEG	POS	POS	NO	NO	NO	0301	0405
295	F	6.6	YES	NEG	POS	POS	NEG	NO	NO	NO	0102	0401
296	F	6.7	YES	POS	POS	NEG	NEG	NO	NO	NO	0402	0402
297	M	12.4	NO	POS	POS	NEG	POS	NO	NO	NO	0101	0301
298	M	13.8	YES	NEG	POS	NEG	NEG	NO	NO	NO	0301	0301
299	F	3.8	NO	NEG	POS	POS	POS	YES	MOTHER	NO	0301	0404
300	M	10.6	NO	POS	POS	POS	POS	NO	NO	NO	03	07

Patient ID	Gender (Female:F; Male:M)	Age at onset (years)	DKA	IAA	GADA	IA2A	ZnT8A	First degree relatives with DM	Parents with DM	Siblings with DM	DRB1_ Allele1	DRB1_ Allele2
301	F	10.3	NO	NEG	POS	POS	POS	NO	NO	NO	0402	0402
302	F	8.7	NO	NEG	NEG	POS	POS	NO	NO	NO	0301	0405
303	M	12.5	NO	POS	POS	POS	NEG	NO	NO	NO	0404	0404
304	M	11.6	NO	NEG	NEG	POS	POS	NO	NO	NO	0301	0301
305	F	6.8	YES	POS	NEG	POS	POS	NO	NO	NO	0301	0404
306	M	8.5	YES	POS	POS	NEG	POS	NO	NO	NO	1303	1404
307	F	1.8	NO	POS	NEG	NEG	NEG	NO	NO	NO	0401	0701
308	F	7.5	NO	NEG	POS	NEG	NEG	YES	MOTHER	NO	0301	0301
309	F	1.0	YES	POS	POS	NEG	NEG	NO	NO	NO	03	03
310	M	8.5	NO	POS	NEG	POS	POS	NO	NO	NO	0301	0301
311	M	12.1	NO	POS	NEG	NEG	POS	YES	FATHER	YES	03	01
312	F	10.4	YES	POS	POS	NEG	NEG	NO	NO	NO	0102	1404
313	F	7.3	NO	NEG	POS	POS	POS	NO	NO	NO	0101	0405
314	M	10.3	YES	NEG	POS	POS	POS	NO	NO	NO	0301	0301
315	F	14.0	NO	NEG	POS	NEG	NEG	YES	FATHER	NO	0301	0301
316	M	12.5	NO	POS	POS	POS	POS	NO	NO	NO	0301	0402
317	M	7.1	YES	NEG	POS	NEG	POS	NO	NO	NO	0102	0801
318	M	12.3	YES	POS	POS	POS	POS	YES	MOTHER	NO	01	04
319	M	11.2	NO	POS	POS	POS	POS	YES	NO	YES	0403	1601
320	M	12.7	NO	NEG	POS	POS	POS	NO	NO	NO	0101	0301
321	M	11.8	NO	NEG	POS	NEG	POS	NO	NO	NO	0301	1302
322	F	10.6	YES	NEG	POS	NEG	NEG	NO	NO	NO	0301	0701
323	F	8.8	NO	POS	POS	POS	POS	NO	NO	NO	0301	0301
324	F	8.4	YES	NEG	POS	NEG	NEG	NO	NO	NO	0301	0301
325	M	12.8	YES	POS	POS	NEG	NEG	NO	NO	NO	0401	0401
326	M	6.8	NO	POS	POS	POS	NEG	NO	NO	NO	0405	0701
327	M	5.0	YES	POS	POS	POS	NEG	NO	NO	NO	0301	0901
328	M	9.9	NO	POS	POS	POS	POS	YES	MOTHER	NO	0405	1301
329	F	11.5	YES	POS	POS	NEG	POS	NO	NO	NO	03	04
330	F	9.3	YES	POS	POS	POS	NEG	YES	FATHER	NO	0404	0701
331	M	4.0	NO	POS	POS	POS	POS	YES	MOTHER	NO	0301	0405
332	M	9.5	YES	POS	POS	POS	POS	NO	NO	NO	03	07
333	M	12.8	YES	POS	POS	POS	POS	NO	NO	NO	0701	1102
334	F	7.9	NO	POS	POS	POS	POS	NO	NO	NO	0301	0301
335	F	12.4	NO	POS	POS	POS	POS	NO	NO	NO	0401	1302
336	F	10.8	YES	NEG	POS	NEG	NEG	NO	NO	NO	0301	0401
337	M	13.3	NO	POS	POS	POS	POS	NO	NO	NO	04	13
338	M	9.9	YES	POS	POS	POS	POS	NO	NO	NO	0401	0402
339	F	6.8	NO	POS	POS	NEG	NEG	NO	NO	NO	1101	1401
340	M	1.9	NO	POS	NEG	POS	NEG	YES	FATHER	NO	0301	0701
341	M	13.7	YES	POS	POS	POS	POS	NO	NO	NO	0401	0701
342	M	14.8	NO	NEG	NEG	POS	NEG	NO	NO	NO	0301	0404
343	M	9.6	NO	POS	POS	NEG	NEG	NO	NO	NO	13	01
344	M	1.4	NO	POS	NEG	NEG	NEG	YES	FATHER	NO	0405	0701
345	F	14.8	YES	NEG	POS	NEG	NEG	NO	NO	NO	01	03
346	M	6.1	YES	POS	POS	POS	NEG	NO	NO	NO	0301	0402
347	F	7.8	YES	NEG	POS	NEG	POS	NO	NO	NO	0301	0402
348	M	1.3	YES	POS	NEG	NEG	NEG	NO	NO	NO	0301	0301
349	M	9.4	NO	NEG	POS	POS	POS	NO	NO	NO	0301	0401
350	M	12.3	YES	POS	POS	POS	POS	NO	NO	NO	03	04

Patient ID	Gender (Female:F; Male:M)	Age at onset (years)	DKA	IAA	GADA	IA2A	ZnT8A	First degree relatives with DM	Parents with DM	Siblings with DM	DRB1_ Allele1	DRB1_ Allele2
351	F	4.4	NO	POS	NEG	POS	POS	YES	MOTHER	NO	0301	0405
352	M	13.3	YES	POS	POS	POS	POS	NO	NO	NO	0301	0403
353	M	6.0	YES	POS	POS	POS	POS	NO	NO	NO	0301	0404
354	M	13.2	NO	POS	POS	NEG	NEG	NO	NO	NO	01	03
355	F	5.3	YES	POS	NEG	POS	POS	YES	MOTHER	NO	09	16
356	M	13.2	YES	POS	POS	POS	POS	YES	FATHER	NO	0301	0402
357	F	7.2	NO	POS	NEG	POS	NEG	NO	NO	NO	0301	0405
358	M	12.7	YES	NEG	NEG	POS	POS	NO	NO	NO	0301	0301
359	M	6.0	YES	POS	POS	POS	POS	NO	NO	NO	0101	0401
360	F	7.8	YES	NEG	POS	NEG	POS	YES	MOTHER	NO	0404	0405
361	F	12.0	NO	NEG	NEG	POS	POS	NO DATA	NO DATA	NO DATA	0301	1302
362	F	12.1	NO	POS	POS	NEG	POS	NO	NO	NO	0301	0404
363	M	12.8	NO	POS	POS	NEG	POS	NO	NO	NO	0301	0405
364	F	6.8	YES	POS	POS	POS	NEG	NO	NO	NO	0301	0404
365	M	9.3	YES	POS	NEG	POS	POS	YES	MOTHER	NO	0401	0402
366	M	1.3	YES	POS	POS	POS	NEG	NO	NO	NO	0102	0405
367	M	9.3	NO	POS	NEG	NEG	POS	NO	NO	NO	0301	0404
368	M	12.8	NO	POS	POS	POS	NEG	NO	NO	NO	0301	0404
369	M	9.8	NO	POS	POS	POS	POS	NO	NO	NO	0301	0701
370	M	4.5	NO	NEG	POS	POS	NEG	NO	NO	NO	0301	0401
371	F	4.5	NO	POS	POS	POS	POS	NO	NO	NO	0301	0402
372	F	10.3	NO	POS	POS	NEG	NEG	NO	NO	NO	0301	0405
373	F	10.1	NO	POS	POS	NEG	POS	NO	NO	NO	0401	1601
374	M	4.3	YES	POS	POS	POS	NEG	NO	NO	NO	0301	1104
375	M	14.8	NO	POS	POS	POS	POS	YES	NO	YES	0301	0801
376	M	10.8	YES	POS	NEG	POS	POS	NO	NO	NO	0301	0410
377	M	12.0	YES	NEG	POS	POS	POS	NO	NO	NO	0404	1302
378	M	3.8	NO	NEG	POS	POS	POS	NO	NO	NO	0301	1303
379	M	2.5	YES	POS	POS	POS	POS	NO	NO	NO	0301	0403
380	M	6.8	YES	POS	NEG	POS	NEG	NO	NO	NO	0101	0405
381	F	11.8	YES	NEG	NEG	NEG	POS	NO	NO	NO	0301	0404
382	M	10.2	YES	POS	POS	POS	POS	NO	NO	NO	0301	1501
383	M	13.4	YES	POS	NEG	POS	POS	NO	NO	NO	0701	0801
384	F	13.1	NO	POS	POS	NEG	NEG	NO	NO	NO	0301	0402
385	M	10.0	NO	NEG	POS	NEG	NEG	NO	NO	NO	03	03
386	F	1.6	YES	POS	POS	NEG	NEG	NO	NO	NO	0301	0405
387	M	8.8	NO	NEG	POS	POS	POS	NO	NO	NO	0405	1502
388	F	10.6	YES	POS	NEG	POS	NEG	NO	NO	NO	0405	0801
389	F	12.6	NO	POS	POS	POS	POS	YES	FATHER	NO	0404	0802
390	M	13.3	NO	POS	NEG	POS	NEG	NO	NO	NO	0301	1302
391	M	13.7	NO	NEG	NEG	POS	NEG	NO	NO	NO	0301	0403
392	M	3.4	NO	POS	NEG	POS	POS	NO	NO	NO	04	13
393	F	9.5	NO	POS	POS	POS	POS	NO	NO	NO	0401	0701
394	F	12.5	NO	NEG	POS	POS	POS	NO	NO	NO	0301	1301
395	F	8.5	NO	POS	POS	NEG	POS	NO	NO	NO	0701	0801
396	M	2.1	NO	NEG	NEG	POS	POS	NO	NO	NO	0301	0404
397	F	7.2	NO	POS	POS	POS	POS	NO	NO	NO	0301	0404
398	F	4.4	YES	NEG	NEG	POS	POS	NO	NO	NO	0405	1301
399	F	4.4	YES	POS	POS	POS	POS	NO	NO	NO	0301	0402
400	F	10.7	NO	POS	POS	POS	NEG	NO	NO	NO	0301	0405

Patient ID	Gender (Female:F; Male:M)	Age at onset (years)	DKA	IAA	GADA	IA2A	ZnT8A	First degree relatives with DM	Parents with DM	Siblings with DM	DRB1_ Allele1	DRB1_ Allele2

F: Female. M: Male; DKA: Diabetic Ketoacidosis at diagnosis according to ISPAD [10]; 1st degree relatives: parents and/or siblings; HLA-DRB1 risk alleles are defined based on the report [11]: 0 risk alleles (no DR3 no DR4); 1 risk allele (DR3 or DR4); 2 risk alleles (DR3/3, DR4/4 or DR3/4). In our cohort, DRB1*0403 allele does not confer susceptibility to T1D so it is excluded from DR4 alleles group in subsequent analyses.
